# Social media and colorectal cancer: A systematic review of available resources

**DOI:** 10.1371/journal.pone.0183031

**Published:** 2017-08-23

**Authors:** Gianluca Pellino, Constantinos Simillis, Shengyang Qiu, Shahnawaz Rasheed, Sarah Mills, Oliver Warren, Christos Kontovounisios, Paris P. Tekkis

**Affiliations:** 1 Department of Colorectal Surgery, Royal Marsden Hospital, London, United Kingdom; 2 Department of Colorectal Surgery, Chelsea and Westminster Hospital, London, United Kingdom; 3 Department of Surgery and Cancer, Imperial College, London, United Kingdom; University of California Los Angeles David Geffen School of Medicine, UNITED STATES

## Abstract

**Aim:**

Social media (SM) can provide information and medical knowledge to patients. Our aim was to review the literature and web-based content on SM that is used by Colorectal Cancer (CRC) patients, as well as surgeons’ interaction with SM.

**Method:**

Studies published between 2006 and 2016 were assessed. We also assessed the impact of several hashtags on Twitter with a freeware (Symplur).

**Results:**

Nine studies were included assessing Twitter (78%), Forums/Cancer-survivor networks (33%), and Facebook (22%). Aims included use of SM by CRC patients (67%), cancer-specific usage of SM with different types of cancer (44%), content credibility (33%), and influence in CRC awareness (33%). Prevention was the most common information that CRC patients looked for, followed by treatment side-effects. Only 2% of CRC SM users are doctors. SM use by colorectal consultants was suboptimal. Only 38% of surgeons had a LinkedIn account (most with less than 50 connections), and 3% used Twitter. A steep increase of tweets was observed for searched Hashtags over time, which was more marked for #ColonCancer (+67%vs+38%, #Coloncancer vs #RectalCancer). Participants engaged with colon cancer increased by 85%, whereas rectal cancer ones increased by 29%. The hashtag ‘#RectalCancer’ was mostly tweeted by colorectal surgeons. The official twitter account of American Society of Colorectal Surgeons (@fascrs_updates) was the most active account.

**Conclusion:**

CRC patients and relatives are increasingly engaging with SM. CRC surgeons’ participation is poor, but we confirm a trend toward a greater involvement. Most SM lack of authoritative validation and the quality of shared content still is largely anecdotic and not scientifically evidenced-based. However, SM may offer several advantages over conventional information sharing sources for CRC patients and surgeons, and create connections with mutual enrichment.

## Introduction

Social Media (SM) have revolutionized medical practice. Most people currently hold an account in available SM, and the use of SM by scientific medical societies and organisations is rapidly growing over the last years.

It has been estimated that over 1.5 billion people use SM, and 80% of them are interacting actively on, at least, a monthly basis [1]. The most used SM by surgeons and patients include Twitter, Facebook, and LinkedIn, which range from private/social to business-oriented contents. Other forms of SM that can be relevant for doctors and patients include web forums and channels to share videos and pictures (e.g. YouTube and Instagram).

SM allows a variable degree of interactions, and has the potential to provide patients with information concerning their disease. At the same time, scientists can benefit from SM in many ways. However, concerns have been raised about the scientific soundness of available content, and doctors’ involvement in SM interactions and development.

Several studies have investigated the impact of SM and their relevance to specific patient populations, but no research has ever reported on the value of SM in colorectal cancer (CRC).

The aim of this study is to address how patients diagnosed with CRC interact with SM, and to assess the engagement of CRC surgeons, by means of a systematic review of the literature and online SM analytic software.

## Materials and methods

### Inclusion criteria

- Studies and reviews published during the last ten years on SM use in CRC patients.
- Studies where CRC patients were clearly identifiable and/or subgroup analyses were satisfactorily reported.
- At least one SM, including Twitter, Facebook, LinkedIn, and online forums had to be reported on the studies.
- Papers reporting on the use of SM by CRC surgeons.
- Only full-text articles were included.

### Exclusion criteria

- Studies were not included if they analysed only platforms or digital content that did not allow interactions among users.

### Data search

Available data from all studies published between January 2006 and December 2016 were evaluated for inclusion. We decided to include only studies published in the last ten years because the use of SM has been spreading recently. We searched the PubMed database, the Science Citation Index Expanded, the Cochrane Library, and we used the Google search engine. Free text words, keywords, and medical subjects headings (MeSH) used were: “colorectal cancer”, “social media”, “surgery”, “twitter”, and “facebook”. The limits used were publication date between 2006 and 2016. We used the cross-referencing and related articles functions. The last search was run on January 2017.

We also assessed the impact of the #colorectalcancer hashtag on Twitter, by using free online software (Symplur). In order to seek for differences between colon and rectal cancer, we also analysed and compared the performance of the two different hashtags: #coloncancer and #rectalcancer.

### Aims

Primary: Usage of SM and their impact in CRC patients.

Secondary: Usage of SM and perspectives from CRC surgeons, specifically the yield of SM has added to conventional ways of sharing knowledge, acquiring skills, and caring for patients.

Impact and trend of hashtags in CRC surgery and treatment over the last two years.

This review had qualitative outcomes, but we made an effort to obtain quantitative measures as well.

### Data extraction

All selected publications were read by two authors (GP, CK). Data of interest: years of study; year of publication; type of SM assessed; patient/subject population; main findings; main concerns and limitations.

Some SM use *hashtags* to aggregate themes pertaining to the same topic. A hashtag consists of a word or a sentence (without blank spaces) preceded by the *hash* character *(*#). By searching the word that is used as hashtag (e.g. #coloncancer), users will get access to all of the contents in which the word is tagged. Some resources are available online, which use algorithms to obtain information on the outreach and impact of hashtags in SM. Symplur) is a healthcare social media analytics company that provides a free online tool, the Healthcare Hashtag Project, aimed at connecting patients and doctors to contents that are available on Twitter, based on several medical hashtags [2]. Symplur holds two Twitter accounts (@symplur and @healthhashtags), of which one is dedicated to the Healthcare Hashtag Project, and it also offers data concerning usage and performance of health hashtags (http://www.symplur.com/healthcare-hashtags/diseases/). We compared the outreach and features of the three hashtags (#colorectalcancer, #coloncancer, and #rectalcancer) in two consecutive, 6-month timeframes (July 2015 to December 2015; January 2016 to July 2016). We compared number of tweets, overall impressions, participants, and influencers.

## Results

**Fig 1:**
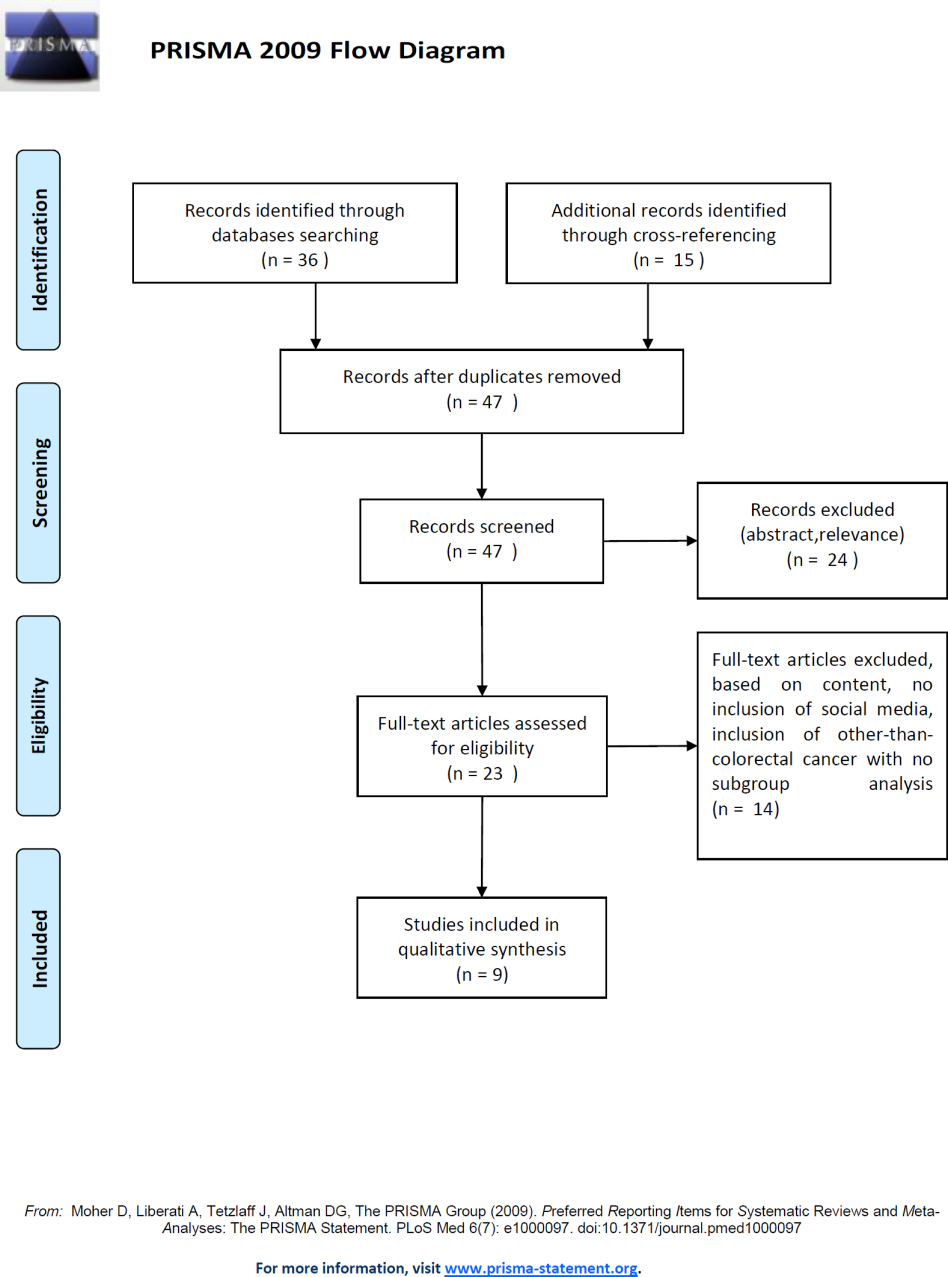
Flow-chart of study selection for inclusion in the analysis.

The initial search identified 36 papers on PubMed, which increased up to 51 after including cross-referenced sources. We assessed 23 full-text papers. A total of 14 were removed either because they were reporting on SM which did not allow interactions or they included non-CRC patients without any sub-analyses. Hence, 9 studies [3–11] dealing with CRC patients met the criteria and were included (Table 1). The algorithm of study selection is reported in Fig 1.The PRISMA checklist was followed (S1 Checklist).

**Table 1:** Studies included in the review.

	Author	Year	Social Media	Purpose	Findings	Concerns
**A**	De la Torre-Dὶez[3]	2012	Facebook	A) use of SM in chronic diseases	Facebook 62% / Twitter 31.7%	Suboptimal doctor involvement
			Twitter	B) Credibility of information	26% prevention issues	
	Beusterien [4]	2013	2 forums for cancer patients	Impact of CRC in patient’s forum	Participants: • 83% patients / 17% relatives • 76% female gender • Mean age 49 years	• Lack of formal knowledge • Increased anxiety and uncertainty
					Topic: • 62% side effects	
	Cutrona [5]	2013	Facebook	Peer-to-Peer chat for cancer screening	Facebook 12.3%	CRC: most sharing experiences on screening via email (32%)
			Twitter		Email 12%	
			Other		Twitter 4.8%	
	Portier [6]	2013	Cancer survivors network	Topics & Sentiment analysis	Negative initial emotion predict sentiment change	Lack of automated, reliable tools to identify patients at risk
	Tsuya [7]	2014	Twitter	Cancer patients usage	• CRC do share info via SM • Useful information for doctors • Different issues for each cancer	Analysis and content are strongly influenced by other media (e.g. television)
	Park [8]	2016	Twitter	Credibility of information	• 76119 tweets • 90% individual users	Only 2% of individual users are doctors
	Xu [9]	2016	Twitter	Frequency of discussion according to cancer, race, gender	Increased tweeting and exposure during “awareness months”	CRC receive least Twitter attention
	Crannell [10]	2016	Twitter	A) content of tweets by the US cancer patients;	Patients express themselves openly on SM and happiness is influenced by the type of cancer	CRC receive least Twitter attention
				B) average happiness of patients		
	Lee [11]	2016	Twitter	CRC Twitter content and transmissibility of awareness campaign in Korea	Most tweets were spam and commercial	Transmissibility of the awareness campaign was questionable. Public health institutions and organizations must be involved in SM
**B**	Mc Donald [12]	2015	LinkedIn	Uptake and use by CRC surgeons in the UK	37% LinkedIn	UK consultants poorly engaged with SM
			Twitter		3.1% Twitter	

A: Patients; B: Surgeons; CRC: colorectal cancer; SM: social media

Only one paper reported on surgeons’ perspective concerning SM in CRC [12] (Table 1).

### Primary aim

#### Patients

All studies were published between 2012 and 2016 (44.4% in 2016) Facebook was assessed in 22.2% [3, 5] of them, Twitter in 77.8% [3, 5, 7–11], and Forums/Cancer survivor networks in 33.3% [4–6]. The most used and investigated SM before 2014 seemed to be Facebook, whereas studies published afterwards have focused on Twitter.

Aims of the studies included the use of SM by CRC patients (66.7%) and cancer-specific usage of SM in patients with different types of cancer (44.4%), the credibility of available content (33.3%), and the influence of SM in CRC awareness and screening (33.3%).

Most patients with CRC use SM to get information and increase disease awareness. Age does not seem to influence the use of SM [4]. One study found that up to 17% of patients’ relatives may join forums dedicated to cancer patients [4]. Prevention was the most common information that CRC patients looked for, followed by side effects of treatment, which captured the attention of up to 62% of patients in one study [5, 7]. Patients shared their experience and their feelings, and studies agree that this can have a beneficial effect for patients [3, 6, 7, 10], but it may also provide doctors with a useful insight on patients’ needs.

There are differences among different kinds of cancer and patient’s usage of SM. Even if CRC patients do use SM, CRC seems to be poorly represented, especially on Twitter, as compared with other cancers (e.g. breast or lung) [7, 9, 10]. One study [5] found that CRC patients are more likely to share their experience concerning CRC screening via private email rather than SM. This proportion is the highest compared with non-CRC patients, exceeding 30%.

Scientific credibility represents an issue in all of the studies assessing this aspect. Shared information is strongly influenced by mass media (e.g. television), and doctor involvement in either SM or forums is scanty. Ninety percent of the sources of information on CRC available in SM are shared by individual users, of which only 2% are doctors. The lack of knowledge, and the uncertainty related to it, have negative effects on patients’ feelings and can worsen their anxiety and negative feelings, that are strictly connected with CRC management [6, 11].

Even if CRC is receiving less attention than other cancers, SM have beneficial effects on awareness and can increase the number of patients attending screening. Again, the contribution of other media is relevant, because exposure seems to be higher during ‘awareness campaign’ time. Indeed, SM has broadened the reach of awareness campaign [9].

### Secondary aims

#### Colorectal surgeons

Our literature review has not generated any paper addressing the use of SM by colorectal surgeons. A survey [12] from the UK investigated General Medical Council (GMC) registered colorectal surgeons’ access to SM. Compared with other specialties, the use of SM by colorectal consultants in the UK was suboptimal, and the most used SM was LinkedIn. Only 38% of surgeons had a LinkedIn account, and 3% used Twitter. Nearly 65% of Twitter users also had a LinkedIn account. Engagement was poor, as most surgeons had less than 50 connections on LinkedIn.

#### Hashtags

**Fig 2:**
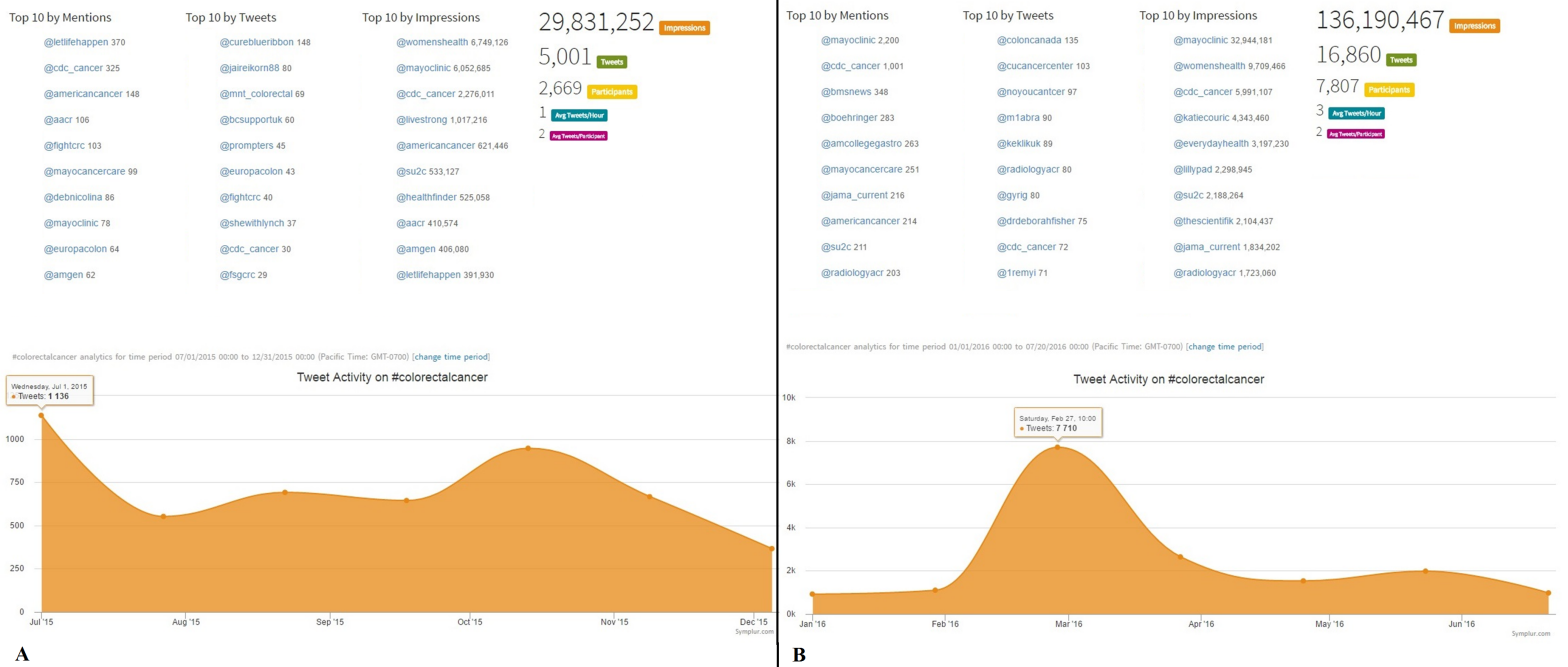
**Comparison of the performance of the hashtag *#colorectalcancer* in two different 6-month time-frames: July 2015 –December 2015 (2A) and January 2016 –July 2016 (2B).** The number of tweets reached 16860 in the last months, compared with the 5001 recorded in the previous period. Participants in discussions including the hashtag were three times higher during the last months, with over 136 million impressions (almost 5 times more). The Mayo Clinic official account was the first in both Mentions and Impressions over the last months, followed by medical associations and Institutions. It is worth noting that eight out of ten accounts which were in the top list in terms of mentions during the last months belong to renewed CRC Institutions and Centres (e.g. Center for Disease Control and Prevention, CDC @cdc_cancer, Mayo Clinic @mayoclinic and @mayocancercare), Scientific Societies (American College of Gastroenterology ACG @amcollegegastro), and Scientific Journals (e.g. JAMA, @jama_current), whereas one belongs to a fundraising organization (Stand Up to Cancer @su2c) another one to a Pharmaceutical Company (Boehringer Ingelheim @boehringer). This suggests that, irrespective of who are the most active tweeters, the quality of the tweets can be Scientifically relevant and true[8]. (reprinted from Symplur LCC under a CC BY license, with permission from Thomas M.Lee, Co-Founder, Symplur, LLC, original copyright 2016, http://www.symplur.com).

Concerning #colorectalcancer, the number of tweets tripled from 5001 to almost 17000 (Fig 2) in the two time frames of the current study. Similarly, impressions during the last months went up to 136 million compared with almost 30 million in the previous timeframe. One of the largest CRC Centres was the most represented in terms of mention and impressions during the last months of the current study, whereas in the previous months the top positions were held by individual accounts or associations (Fig 2). During the second timeframe, the highest number of tweets (n = 7710) was reached during March 2016, in conjunction with the CRC awareness month promoted by the American Society of Colon and Rectal Surgeons (ASCRS). During the previous months, the highest number of tweets (n = 1136) was observed in July 2015, presumably being associated with the Ethnic Minority Cancer Awareness Month (EMCAM) 2015.

**Fig 3:**
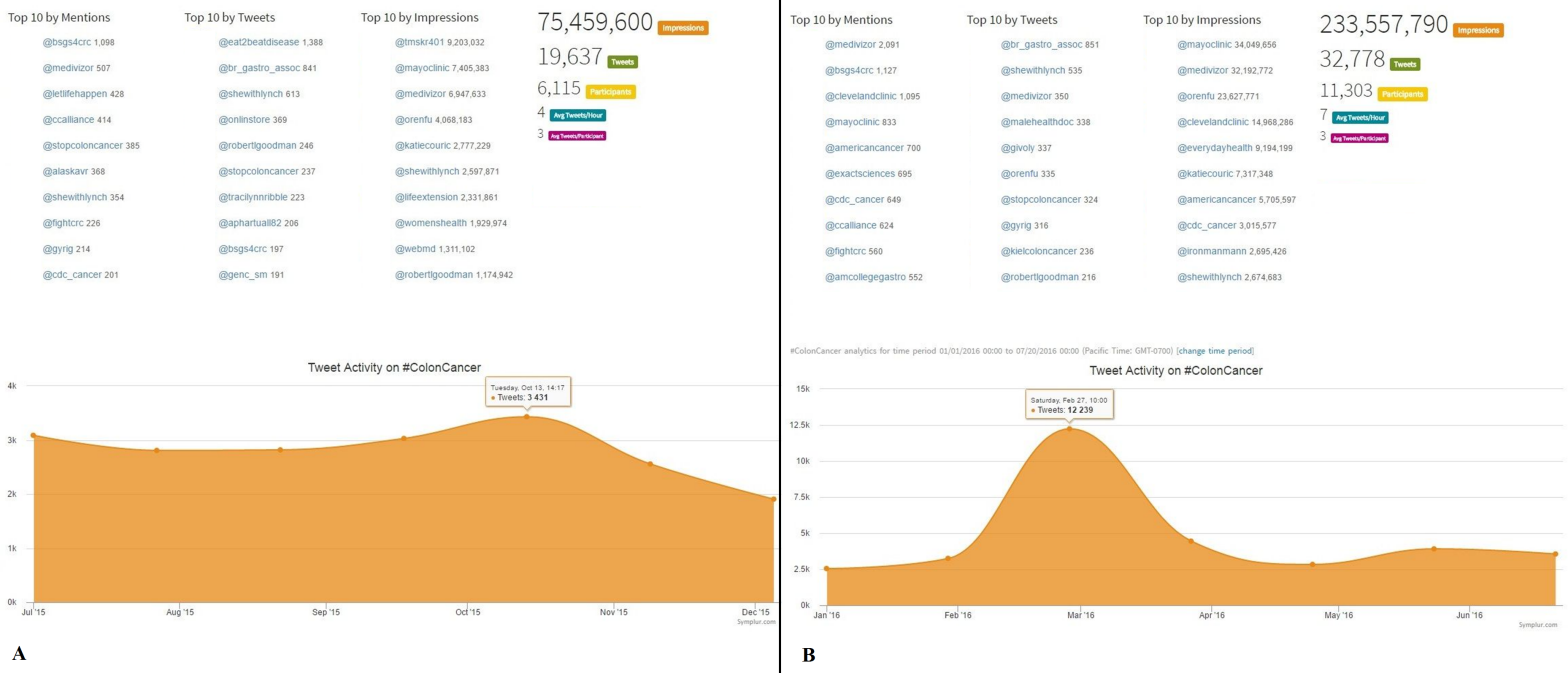
**Comparison of the performance of the hashtag *#coloncancer* in two different 6-month time-frames: July 2015 –December 2015 (3A) and January 2016 –July 2016 (3B).** The number of tweets exceeded 32000 in the last months, compared with the 19637 recorded in the previous period. Impressions were three times higher over the last months, and involved participants almost doubled. In both time-frames, the most active tweeters included mostly private accounts and associations, but the latter increased in the last months. In addition, over the last months, the accounts of Scientific Institutions and Associations were the most mentioned and those with highest number impressions (reprinted from Symplur LCC under a CC BY license, with permission from Thomas M.Lee, Co-Founder, Symplur, LLC, original copyright 2016, http://www.symplur.com).

**Fig 4:**
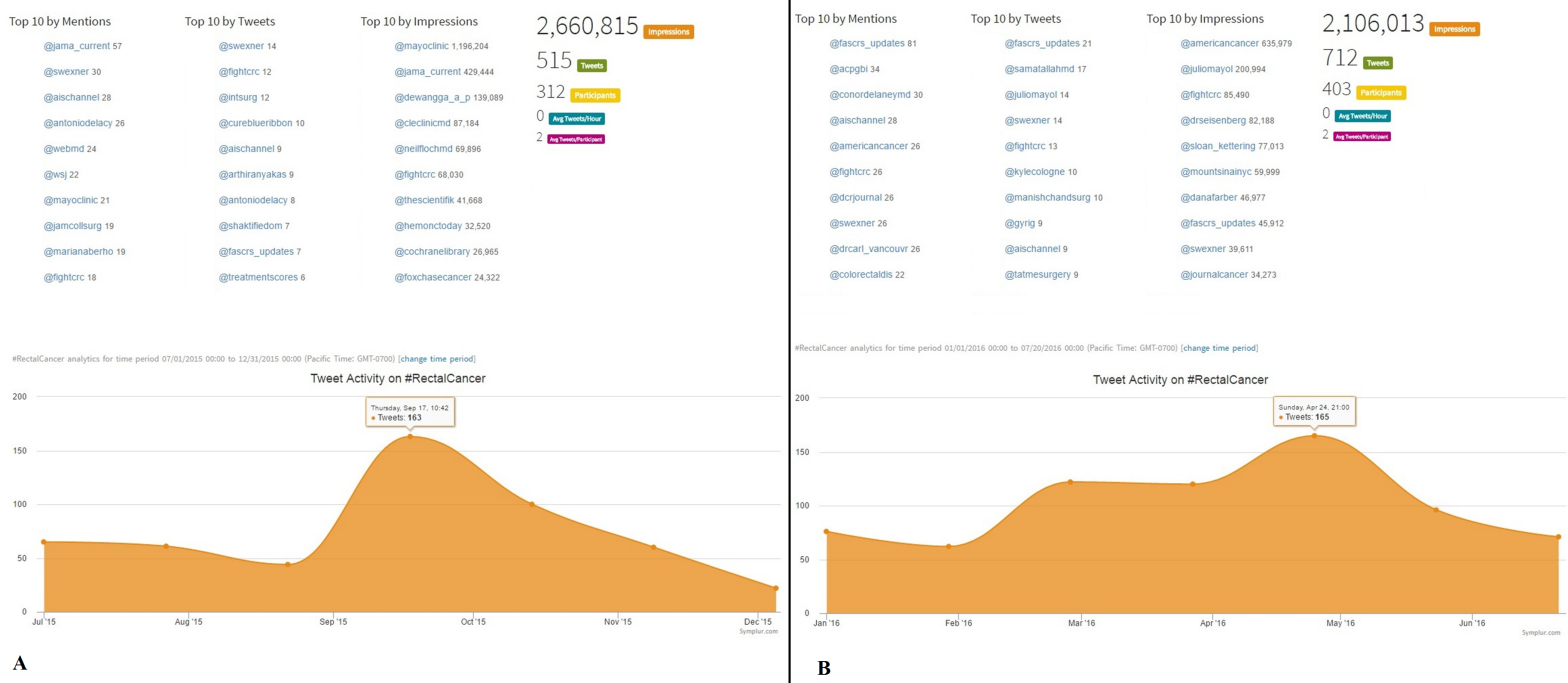
**Comparison of the performance of the hashtag *#rectalcancer* in two different 6-month time-frames: July 2015 –December 2015 (4A) and January 2016 –July 2016 (4B).** Even for this hashtag the number of tweets increased (+200), but the overall impressions slightly decreased (-560000). Notably, the most active tweeters were experts in CRC and CRC Scientific Association in both time-frames. This can be justified by a reluctance of patients and individual users in sharing their experiences on rectal cancer, potentially due to shame of the investigations needed to obtain diagnosis (e.g. digital rectal examination) and signs of disease (e.g. rectal bleeding). There is room for improvement for a true holistic care of CRC patients, embracing their social life and feelings[4, 6]. (reprinted from Symplur LCC under a CC BY license, with permission from Thomas M.Lee, Co-Founder, Symplur, LLC, original copyright 2016, http://www.symplur.com).

Concerning the two different conditions (colon cancer and rectal cancer), a steep increase of tweets was observed in both hashtags over time, which was more marked for #ColonCancer (an increase of 67% and 38% for #Coloncancer and #RectalCancer, respectively). The number of participants engaged with colon cancer increased from 6115 to 11303 (85%), whereas that of rectal cancer increased from 312 to 403 (29%). The #RectalCancer hashtag was mostly tweeted by individual colorectal surgeons, but the official twitter account of a colorectal society (ASCRS, @fascrs_updates) was the most active account during the second timeframe (Figs 3 and 4). Consistent with an increased engagement, and perception that SM are an important tool to share information related to colon cancer (#coloncancer), the top 10 trends by mention over the second timeframe includes more Institutions who are treating CRC (@clevelandclinic, @mayoclinic) and scientific societies (@amcollegegastro), with @mayoclinic being the account holding the first position in ‘impressions’ (over 34 million). The changes in #rectalcancer were even more striking, as @fascrs_updates were the first account in terms of number of Tweets during the second timeframe, followed by individual experts in the field. However, the number of impressions for #rectalcancer were lower in the last months compared with the previous ones (Fig 4).

## Discussion

SM has completely changed the way people communicate and share experiences, and patients and surgeons have been involved in this profound change.

### Colorectal cancer patients’ perspective

Patients suffering from cancer have been reported to use several SM, for different reasons. Many SM are currently available and each of them has specific features allowing users to interact with each other. Some SM, such as YouTube, permits the users to share even long videos on either public or private profiles. From our systematic review, we found that most CRC patients used Facebook, Twitter, or both, whereas a lower proportion of them prefer more confidential SM, such as dedicated cancer online forums or even private emails. Facebook is the most used SM worldwide. By 2015, more than 1.59 billion active users have been registered [13]. It allows to create a personal profile as well as Institution/Organization pages, which can upload information, comments, and share material. However, Facebook has been temporarily or permanently blocked in many Countries, for various reasons.

Twitter is another SM that has been increasingly used by colorectal surgeons and patients. Users are allowed to share with ‘followers’ sentences not exceeding 140 characters, links, pictures, and short videos. Recently, the possibility of posting polls (questions with the possibility of choosing between a limited numbers of answers) has been added. Users can re-tweet and like the tweets of other users, and reply to them with ‘@’ function. *Hashtags* are specific to Twitter and other SM, and have make it possible to obtain information on a specific topic from across several SM at the same time, and are probably the feature of SM which is most likely to be irreplaceable. Nevertheless, the scientific content of Twitter needs to be carefully monitored.

Interactivity is one of the most relevant opportunities that SM offers CRC patients (https://hbr.org/2011/12/using-social-networks-to-impro). It is interesting that SM can also help patients’ relatives share their feelings with those who are going through the same experience [4]. CRC patients often use SM to obtain information about screening and prevention [3], and many of them are scared by the side effects of treatments [4]. From this perspective, the interactive nature of SM could overcome cancer-specific issues in CRC patients but, at the same time, can be limited by some aspects of CRC itself.

CRC patients need to share their feelings [4, 6], and they can provide invaluable support to other persons at risk of developing CRC [2, 6, 7]. Wong et al. [14] conducted a survey on over two thousand subjects aged ≥ 50 years in Singapore to identify factors associated with CRC screening uptake. They found that most of them were scared by the implications of being diagnosed with CRC, in terms of either suffering (90%) or treatment costs (83%). Other studies confirmed that the fear to be unable to afford the expenses of treatment was associated with lower adherence to CRC screening guidelines and with lower detection of early CRC [15]. Notably, few individuals have been reported to undertake screening following the advice of their doctors [14]. SM involvement by CRC patients can provide at risk people with reassurance related to their economic or treatment-related concerns.

It can easily be understood that there is a larger audience of SM than in any other way of sharing information, and evidence supports the benefits of SM in increasing CRC awareness and screening uptake [9, 14]. However, SM can also act as a tool to actually prevent CRC. Hawkes et al. [16] assessed the efficacy of a telephone-delivered intervention in reducing the risk factors for CRC by modifying life-style and behaviour of individuals at risk. They found that participants obtained improvements in level of physical activity and fibre intake, whilst reducing alcohol consumption, processed meat intake, and their body mass index and waist circumference. At the same time, they had higher scores in both physical and mental items of the health-related quality of life SF-36 questionnaire [16]. We have shown that patients with CRC do use SM to interact, hence their potential as a prevention tool in CRC can be foreseen. SM would act as an amplifier of community-engaged approaches, following the concept of ‘Global Village’ associated with the development of SM themselves [17]. Smith et al. [18] were able to create a cookbook with life-style tips useful in preventing cancer. Their project consisted of 1) the involvement of several experts and potential recipients, and 2) circulation of the final product via SM.

SM can reach an even greater number of CRC patients and individuals at risk and widen the opportunity of cure for patients, due to the so-called ‘snow-balling’ effect [19]. This consists of a chain-referral in which participants’ have discussions and share research studies that can get more people who may be interested, or fits the study criteria, engaged by means of their social interactions.

### Surgeons dealing with colorectal cancer

We found that SM usage by CRC surgeons has been poorly reported in the literature, but a growing number of colorectal surgeons are currently involved in SM. Our study has shown, LinkedIn to be the most used SM by CRC surgeons. Founded in 2002, LinkedIn is a business-oriented SM mainly used for professional networking, which by 2015 has reached 400 million members in more than 200 Countries [20]. Each user has their own profile in which they can enlist their competences, which can be confirmed by other users. These skills are used by job recruiters, head hunters, and human resources officers to seek for candidates. This platform was found to be the most used SM by single consultant colorectal surgeons of the Association of Coloproctology of Great Britain and Ireland (ACPGBI); this is especially true for those who were registered with the GMC before 1997, probably because of the strictly professional aspect of this SM [12].

Even if the rate of consultant surgeons from the UK using Twitter with a professional account has been reported to be lower than other SM [12], it is more attractive for younger surgeons and it is being more and more used by authoritative Institutions and Scientific Societies, reaching a larger number of surgeons. More importantly, the huge potential of Twitter is suggested by its uptake and the fact that if it only took one year from the time Facebook launched to grow to 50 million users, Twitter reached 50 million in nine months. By contrast, it took radio and television almost 40 and over 10 years to reach the same audience, respectively, whereas it took Internet three years [1]. Hence, we have decided to search the outreach and usage of two hashtags (#coloncancer and #rectalcancer) on Twitter in two different timeframes. Both hashtags showed increased mentions and reached a larger number of participants in the second time-frames. More importantly, the involvement of CRC surgeons and dedicated scientific societies showed an increase as well, suggesting that reliability and credibility of information are likely to be improved. Even if the engagement of CRC surgeons has been reported to be lower than doctors from other specialties, we were able to find that there has been a steep increase of SM usage by colorectal surgeons.

SM can also provide new ways of teaching that can be implemented in available training courses.

The impact of SM on CRC Organizations should not be underestimated. It is well known that SM can bring business benefit for Organizations, up to 90%, that can lead to 20–25% raise of productivity [1]. Twitter posting of published papers or job/training opportunities through the official accounts of scientific societies, journals, and organizations may reach a wider audience than a paper journal. This is relevant in the era of digital communication as we move towards paperless information sharing, and can also make the content of Journals more attractive and more clear by implementing published papers with additional digital content [21]. These resources can be implemented in the digital libraries of Scientific Societies, which can periodically tweet the news. Moreover, SM can reach more people with limited access to resources, and this is reflected by the more rapid growth of SM in low- and middle- income countries, compared with high-income countries [22, 23].

Scientific Societies can post tweets on upcoming meetings, conferences, and training opportunities. The hashtag of the 2013 American College of Surgeons Clinical Congress (#ACSCC13) was posted in 3000 tweets by 200 participants, but these went up to over 15000 tweets with #ACSCC15 in 2015 (http://www.symplur.com/healthcare-hashtags/ACSCC15/analytics/?hashtag=ACSCC15&fdate=01%2F01%2F2015&shour=00&smin=00&tdate=04%2F01%2F2016&thour=00&tmin=00) [23]. A study [24] on delegates attending the Meeting of the Association of Anaesthetists of Great Britain and Ireland found that the hashtag #WSM12 was tweeted to create and disseminate notes and learning points, and to describe attended sessions, to interact with discussions, and encourage speakers. Moreover, Twitter allowed organizers, exhibitors, speakers, and doctors who did not attend the conference to contribute to the information stream, resulting in a potential audience of 3603 people. Hence, the shortcomings of not using hashtags to publicize events can be predicted.

Societies can also publicize training opportunities in CRC surgery and multimodal patient management, and also provide followers with upcoming deadlines for abstract submission and reduced rates fee for conferences.

In relation to CRC journals and related outreach, the interest of readers and scientists is now shifting from the impact factor to dissemination of published content. Twitter represents a valid tool to offer visibility to accepted papers and additional material, which can be cross-tweeted among the SM profile of other journals and societies. For instance, the New England Journal of Medicine may reach 600 000 readers per week, being a top Scientific Journal, but a SM can reach millions of people and be accessed without restrictions [25]. An increasing number of colorectal journals from all over the world are adopting an official Twitter profile to divulgate new published articles, those which are temporarily or permanently free, link to additional digital content, quick posts, and provide news from affiliated societies and journals (e.g. Techniques in Coloproctology @TechColoproctol and Colorectal Disease @ColorectalDis)

### Caveat and potential of social media in colorectal cancer

Suboptimal doctor involvement is the more frequently reported concern in SM involving CRC patients [3, 4, 8]. Park et al. [8] found that 90% of tweeters sharing cancer content are individual users, and only 2% of them are doctors. Even if the study was published last year, we were able to show that CRC surgeons and societies are increasingly being engaged in SM, hence the credibility of shared sources is likely to be higher than previously estimated.

It should be noted that similar limitations have also been reported with other sources of information on CRC. A study from Germany [26] aimed at assessing the reliability of available leaflets and booklets concerning CRC screening. The authors analysed a total of 41 print sources, and found that most were not compliant with evidence-based medical information. Specifically, they lack adequate reporting of literature evidences whereas up to one third of them did not mention any of the harms of CRC screenings. The quality of information was found to be, to some extent, misleading [26]. Therefore, SM has the potential to spread inaccurate information in a rapid fashion. For this reason, physicians need to get involved in CRC SM in order to ensure that accurate information, based on reliable sources, are disseminated. Opposite to print-only material, contents shared on SM can be commented in real time by other users, potentially identifying incorrect information.

Poor expert involvement can also result in SM having a negative effect on patient feelings, as a consequence of lack of actual knowledge. This generates an increased perception of uncertainty concerning CRC treatment and features, leading to anxiety and further escaping from CRC screening and follow-ups. CRC has received less attention than other types of cancers, and this may be related to a sentiment of shame derived from some aspects of the CRC screening and patient assessment (e.g. colonoscopy, PR examination). These are still considered *taboo* by a relevant number of patients. CRC experts and institutional accounts were the most active tweeters and influencers concerning this hashtag. Involving a growing number of patients and surgeons is necessary to gain a step forward in CRC patients/doctors involvement in SM, eventually overcoming these fears.

SM are strongly influenced by other media [7, 9], i.e. CRC tweeting increased during CRC awareness month [9], and hashtag performances reached the highest level of tweeting during similar campaigns. We would underline that this influence is bi-directional, meaning that SM are likely to impact conventional media, further highlighting the need of CRC experts to be involved.

Many SM are mainly used for leisure or personal purposes, and the quality of scientific content is not routinely controlled. As an example, Facebook news feed algorithms have not been revealed and there have been concern about the possibility that it can expose users to new and challenging ideas or insulate user in potentially misleading own beliefs [27].

An invaluable resource of SM in CRC is the connections that they can create between patients and surgeons. Doctors treating CRC patients may obtain useful information on patients’ feelings and emotions, and they can provide them with further care and address otherwise unmet needs [4, 6]. This interaction should be further analysed, aiming at obtaining reliable, objective tools to identify patients actually needing proactive measures or additional/different management [6].

Lastly, authorities such as the GMC [12, 28] [and the American Medical Association (AMA) [12, 29] have developed guidelines for the ethical conduct of doctors using SM, to preserve patient information and privacy [30].

### Future directions and impact of social media on colorectal cancer societies

Besides divulgation of Scientific contents and the above enlisted features, SM like Twitter have additional potential of fostering or facilitating liaisons between societies and, more importantly, between CRC researchers all over the world [31]. The European Society of Coloproctology (ESCP) has launched an additional Twitter profile dedicated to Pan-European Studies promoted by the Society (@ESCP_studies). Twitter has also contributed to the development of a student- and trainee- led collaborative, currently including more than 100 European Universities, EuroSurg (@EuroSurg) [32]. The first study of EuroSurg (EuroSurg-1) has just closed enrolment, and has been supported by ESCP and by the Italian Society of Colorectal Surgery (SICCR) [33]. SM can also provide young colorectal surgeons with clinical and research training opportunities, overcoming country-specific disparities [34] SM has played a central role in disseminating these Collaboratives, suggesting that SM may also be useful in establishing research networks to deliver high quality studies. Recently, the hashtag #colorectalsurgery showed that there is a great interest of colorectal surgeons in SM[31, 35].

Journals, Societies, and Organizations can use Twitter and other SM for ethical purposes, such as fundraising or support of initiatives directed against discrimination. Examples are provided by the Twitter profile of the Royal Marsden Hospital (@royalmarsden) with the fundraising walk to support cancer research (#MarsdenMarch), or by the support of the Journal of American College of Surgeons (@JAmCollSurg) for the campaign against discrimination in Surgery, ‘I look like a surgeon’ (#ILookLikeASurgeon) (https://twitter.com/JAmCollSurg/status/715230021812617216). These aims are part of their life and mission in CRC patient care.

Indeed, platforms like Symplur’s Health Hashtag Project have been developed with the aim of connecting patients and doctors via SM, and ease interactions. We found that hashtag performances have gone through great modifications over time. Specifically, an increasing number of experts and–more importantly–scientific societies and CRC centres are using hashtags and SM. However, this engagement is much more pronounced in US-based societies, whereas institutions from Europe tend to be less engaged. Nonetheless, it can be predicted that a similar involvement will develop over time.

An increasing number of health apps are being developed, designed to help patients coping with their diseases and gaining insights on their conditions. These can be easily accessed, and some are free of charge or do not require subscriptions. Over 7000 health apps were available in 2012 [36] and after three years their number reached 165 000, according to the Institute for Health Informatics [37]. However, the involvement of experts in the development of such apps is suboptimal, alarmingly not reaching 33% [38]. These observations urge CRC surgeons to participate in this innovation [39].

### Study limitations

This study has several limitations. First of all, SM are very rapidly evolving and changing, therefore results may slightly modify over time. Included studies consisted of individual interviews or analyses performed with search engines which may have evolved over time. Similarly, accessibility to SM might have increased. However, the trend towards higher use of SM by CRC patients as well as the potential and shortcomings associated with their use are important issues that need to be timely addressed by the scientific community.

Hashtag activity was obtained by evaluating the “top 10” list obtained freely by Symplur. A detailed tweets evaluation would have obtained more detailed results. Nevertheless, evaluation of hashtag performance was a secondary aim of this manuscript, and it was intended to give a rough overview of CRC engagement with SM. We did not include in our analysis the hashtag #crcsm (colorectal cancer social media), which has been implemented in the Healthcare Hashtag project cancer ontology. Structured hashtags are increasingly being used by physicians, but they might not be easy to understand for patients. In addition, since the use of SM in CRC surgeons still needs to be encouraged, we would have obtained conflicting results.

## Conclusions

An increasing number of CRC patients and relatives are being engaged with SM, seeking information concerning their disease. CRC surgeons’ participation has been reported to be suboptimal, but our data confirm a trend toward a greater involvement in SM.

Most SM lack of authoritative validation and the quality of shared content is still largely anecdotic and not scientifically evidence-based. We suggest that people are still reluctant to share their experience with CRC, especially with rectal cancer.

However, if carefully handled, SM may offer several advantages over conventional information sharing sources for CRC patients and surgeons, and create connections with mutual enrichment.

## Supporting information

S1 Checklist PRISMA checklist for the systematic review. (DOC)
